# From data to decisions: Toward a Biodiversity Monitoring Standards Framework

**DOI:** 10.1073/pnas.2519347123

**Published:** 2026-03-04

**Authors:** Andrew Gonzalez, Tom August, Sallie Bailey, Kyle Bobiwash, Philipp H. Boersch-Supan, Neil D. Burgess, Barnabas H. Daru, Chris S. Elphick, Robert P. Freckleton, Winifred F. Frick, Alice C. Hughes, Nick J. B. Isaac, Julia P G. Jones, Marco Lambertini, Oisin Mac Aodha, Anil Madhavapeddy, E. J. Milner-Gulland, Andy Purvis, Nick Salafsky, William J. Sutherland, Iroro Tanshi, Varsha Vijay, S. Hollis Woodard, David R. Williams

**Affiliations:** ^a^Department of Biology, Quebec Centre for Biodiversity Science, McGill University, Montreal, QC H3A 1B1, Canada; ^b^Group on Earth Observations Biodiversity Observation Network, Montréal, QC H3A 1B1, Canada; ^c^Habitat, Montréal, QC H2T 2A4, Canada; ^d^UK Centre for Ecology and Hydrology, Crowmarsh Gifford, Wallingford OX10 8BB, United Kingdom; ^e^Natural England, Newcastle upon Tyne NE4 7YH, United Kingdom; ^f^Kyle Bobiwash, Department of Entomology, University of Manitoba, Winnipeg, MB R3T 2N2, Canada; ^g^British Trust for Ornithology, The Nunnery, Thetford IP24 2PU, United Kingdom; ^h^UN Environment Programme World Conservation Monitoring Centre, Cambridge CB3 ODL, United Kingdom; ^i^Center for Macroecology, Evolution and Climate, GLOBE Institute, University of Copenhagen, Copenhagen DK-2100, Denmark; ^j^Department of Biology, Stanford University, Stanford, CA 94305; ^k^Department of Ecology and Evolutionary Biology and Center of Biological Risk, University of Connecticut, Storrs, CT 06269; ^l^School of Biosciences, University of Sheffield, Sheffield S10 2TN, United Kingdom; ^m^Conservation Science, Bat Conservation International, Austin, TX 78746; ^n^Department of Ecology and Evolutionary Biology, University of California, Santa Cruz, CA 95060; ^o^School of Biosciences, University of Melbourne, Melbourne, VIC 3010, Australia; ^p^UK Centre for Ecology & Hydrology, Wallingford OX10 8BB, Oxfordshire, United Kingdom; ^q^School of Environmental and Natural Sciences, Bangor University, Bangor LL57 2UW, United Kingdom; ^r^Department of Biology, Utrecht University, Utrecht 3584 CH, The Netherlands; ^s^Nature Positive Initiative Secretariat, Trelex 1270, Switzerland; ^t^School of Informatics, University of Edinburgh, Edinburgh EH8 9AB, Scotland; ^u^Department of Computer Science, University of Cambridge, Cambridge CB3 0FD, United Kingdom; ^v^Department of Biology, University of Oxford, Oxford OX1 3SJ, United Kingdom; ^w^Biodiversity Futures Lab, Natural History Museum, London SW7 5BD, United Kingdom; ^x^Foundations of Success, Bethesda, MD 20816; ^y^Department of Zoology, University of Cambridge, Cambridge CB2 3EJ, United Kingdom; ^z^Small Mammal Conservation Organization, Benin City 300025, Nigeria; ^aa^University of Washington, Seattle, WA 98195; ^bb^Science Based Targets Network, New York, NY 10008; ^cc^Department of Entomology, University of California, Riverside, CA 92521; ^dd^Sustainability Research Institute, University of Leeds, Leeds LS2 9JT, United Kingdom

**Keywords:** indicators, standardization, conservation, biodiversity monitoring

## Abstract

Achieving the goals of the Kunming–Montreal Global Biodiversity Framework (GBF) requires monitoring systems that can transform heterogeneous observations into consistent, decision-relevant knowledge. Yet current biodiversity data are fragmented, uneven in quality, and seldom comparable across space or time. Existing standards such as Darwin Core, Findable, Accessible, Interoperable, and Reusable (FAIR) and Collective Benefit, Authority to Control, Responsibility, and Ethics (CARE) principles provide important foundations, but they do not connect the full chain from field observation to policy reporting. We introduce the Biodiversity Monitoring Standards Framework (BMSF)—a unifying architecture that links ethical principles, standardized data collection, accredited analytical workflows, and transparent reporting into a single auditable “chain of evidence.” The framework’s novelty lies in its tiered and federated design, enabling national agencies, Indigenous knowledge holders, local communities, and private-sector actors to operate under shared principles while maintaining data sovereignty. By integrating Essential Variables, accredited analytical methods, and open-source implementation pathways, the BMSF allows locally generated data to be aggregated into credible, comparable indicators aligned with GBF targets. Concrete application, such as a national forest-connectivity assessment, demonstrates how the BMSF improves reproducibility, transparency, and policy relevance relative to existing approaches. Implemented generally, this framework would convert fragmented monitoring efforts into a coordinated, scalable system capable of tracking and guiding collective progress toward halting and reversing biodiversity loss.

Addressing the global biodiversity crisis requires a coordinated response underpinned by robust and comparable information to monitor change and direct targeted action (1–6). While societies have long-monitored nature, a renewed commitment is needed to provide the evidence required to meet the goals of the Kunming-Montreal global biodiversity framework (GBF). Historically, monitoring has been fragmented, divided among academic hypothesis testing, national priorities, and conservation science focused on local management objectives. None of these approaches were designed to create the consistent workflows required by a single global knowledge framework. Consequently, initiatives like the GBF create an urgent need for standardization to meet the demands of robust, cross-national trend analysis (7, 8). Standardized workflows can deliver the consistent evidence needed to identify threats and attribute the causes of change, providing a reliable foundation for policy, much like standards have for climate and weather monitoring.

Although the collection of biodiversity data has grown exponentially, its use is hampered by heterogeneous coverage and a lack of strategic prioritization (9–12). While foundational standards like Darwin Core (13) and information systems like GBIF and OBIS have improved data access, they only address parts of the chain required to link observations to GBF indicators. The current landscape of monitoring workflows is itself fragmented, with multiple analytical approaches for variable estimation and trend detection (14–17). This lack of end-to-end standardization severely limits the ability to synthesize findings and confidently assess progress toward international targets (8, 17).

The lack of such an integrated framework is not primarily a result of a single technological gap, but rather a reflection of historical political and institutional fragmentation. Unlike climate science, which has long been galvanized by a unified political mandate under the UNFCCC, biodiversity monitoring has been split among disparate national priorities, academic pursuits, and conservation projects, without a compelling, overarching driver for harmonization. Furthermore, until recently, the technological capacity for large-scale data integration, cloud computing, and AI-driven analytics was not mature or accessible enough to make a global framework operationally feasible. Today, these historical barriers are falling.

We argue that the entire monitoring pipeline—from survey design to reporting—needs a framework to support evolving, interoperable standards. These standards should incorporate Indigenous and local knowledge systems, ethics, and end-user needs, while also supporting adaptive improvement. Crucially, this is a call for interoperability and reliability, not homogeneity, which will be beneficial in all contexts where monitoring is used to support investment and conservation action (18). The World Meteorological Organization (WMO) provides a successful model for this end-to-end approach. Its rigorous standards produce policy-relevant essential climate variables (ECVs) that underpin global assessments like those from the IPCC. This structured approach is a strong precedent and is mirrored in how climate data inform corporate, business, and governmental emissions accounting.

We present a general biodiversity monitoring standards framework (BMSF; see *SI Appendix*, Tables S3 and S4 for acronyms used in this article). The BMSF recognizes the reality of a fragmented landscape of methods and standards but rather than advancing a single, rigid protocol, we propose a flexible structure that can harmonize these diverse existing efforts. The BMSF is modular, linking steps that integrate ethical principles like FAIR (19) and CARE (20), promote the use of Essential Variables (21–24) and other suitable data, and endorse standardized protocols. It also promotes accredited analytical workflows that produce reliable indicators with quantified uncertainty, ensuring data can be processed into comparable, high-confidence insights. The framework is designed to be global, inclusive, and adaptable for diverse users—including national governments, business and financial institutions, NGOs, and Indigenous-led initiatives (25–27)—implementing actions under the GBF. It suggests a tiered structure, a community-driven accreditation process, and is envisioned for operationalization through open tools and technology platforms that make sophisticated, standardized analyses accessible worldwide. The implementation of the BMSF is envisioned through a federated model, building on the strengths of existing organizations and observatories.

Box 1. Lessons learned from the Measurement, Reporting, and Verification (MRV) framework for REDD+ (Reducing Emissions from Deforestation and Forest Degradation; GFOI. 2020).

The FAO has supported countries in developing MRV systems for REDD+ (Reducing Emissions from Deforestation and Forest Degradation) to ensure comparable estimates of forest-related greenhouse gas (GHG) emissions and carbon stocks. This information is crucial for accessing results-based payments. As of 2025, 71 countries have registered on the REDD+ web platform. The MRV framework provides a repeatable workflow to monitor carbon stocks and emissions, offering many lessons: (28): first, there was a clear international mandate and framework—the UNFCCC provides the overarching goals, reporting requirements, and verification process, creating a strong incentive for countries to adopt standards. Second, IPCC methodological guidelines are widely accepted endorsedAQ by the UNFCCC, and these guidelines are essential for establishing credible MRV systems and ensuring comparability and transparency across countries. Crucially, the tools needed are free and open source, e.g., FAO’s Open Foris and SEPAL lower the barrier to entry, promote transparency, and facilitate cost-effective and accurate monitoring of forest cover, accelerating the development of National Forest Monitoring Systems. Third, the FAO and partners have invested substantially in training and technical support, enabling countries to build national ownership and expertise. Training programs address both national-level and local-level needs, such as data collection. Last, there has been a phased and iterative improvement of the workflows. Countries can start with simpler methods (Tier 1) and gradually move to higher tiers (Tiers 2 and 3) as their capacity and data improve.

This article is composed of four parts. First, we define the core components of monitoring workflows. Second, we describe the BMSF and its modules. Third, we provide an example of how the framework applies to a connectivity indicator under GBF Target 3. Fourth, we discuss implementation challenges, the need for a federated standard body, and next steps.

## 1. The Core Components of Generalized Monitoring Workflows

A generalized monitoring workflow combines two systematic cyclical processes linking the planning and design of monitoring to its implementation. Monitoring begins with defining clear monitoring objectives and then moves to the planning, design, and resource allocation cycle. This “planning” cycle informs the “do” cycle of collection and quality-assured processing of observational data and analyses, translating this information into predefined and selected biodiversity indicators and other communication tools for reporting. This culminates in an assessment of progress toward the initial objectives to inform adaptive management and decision-making (29).

**Fig. 1.**
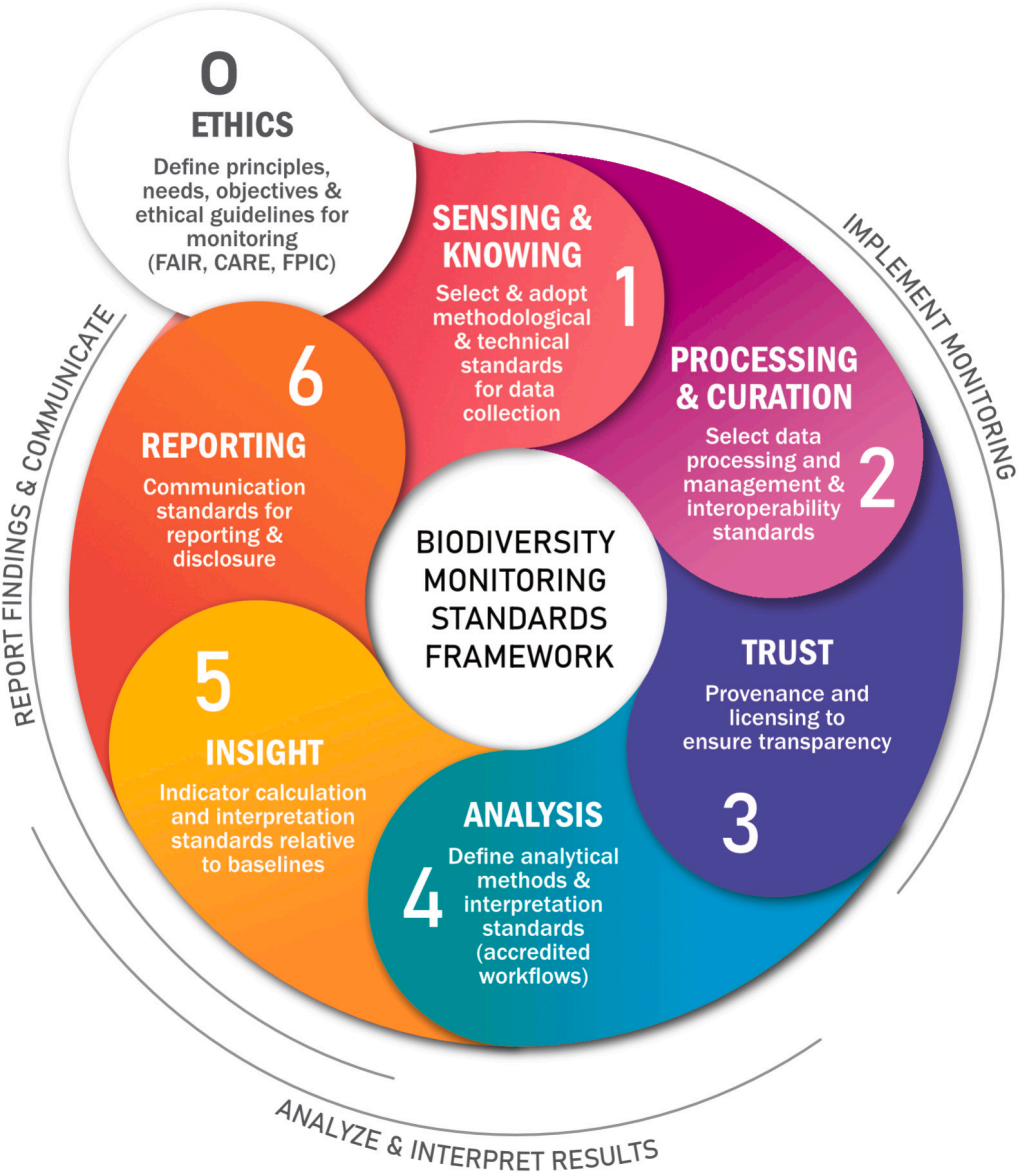
The biodiversity monitoring standards framework is a linked sequence of steps defined by the standards that each step adopts within a set of well-defined needs, principles, and ethical guidelines (*Center*). Every monitoring workflow would achieve an overall standard rating (potentially tiered) based on the standards adopted in each step. The monitoring workflow is implemented (*Right*) once the necessary resources, human capacity, technologies, and data architectures have been adopted. A tiered approach allows countries to start with simpler methods and progressively adopt more sophisticated ones as capacity grows. These standards need not be costly or infeasible to adopt, but they do need to be defined a priori to create trust and confidence in the trends reported.

When viewed at the highest level, we see seven interacting steps that are common to all biodiversity monitoring cycles where standards are necessary (Fig. 1). The cycle starts (step 0) with Foundational Principles & Ethics that identify needs and guiding values, which leads into the cycle in which standards are set for 1) data collection (the “Sensing and Knowing” step); step 2) data processing and management (the “Curation” step), step 3) provenance and licensing (the “Trust” step); step 4) analytical methods and interpretation (The “Analysis” step); step 5) indicator calculation and interpretation (the “Insight” step); and finally 6) reporting and disclosure (the “Reporting” step). Below we discuss each of these steps in turn and then interlink them in a cycle that comprises the BMSF (see *SI Appendix*, Table S2 for a typology of the terms used below).

### 1.1. Step 0: The “Ethics” Step: Foundational Principles and Ethics.

This critical initial step (Step 0, Fig. 1) establishes the overarching needs, values, and guiding principles for all subsequent biodiversity monitoring activities. It defines the “why” and “how” monitoring could be conducted in an ethical, equitable, and just manner. Crucial here is the articulation of how the data will be used and by whom. In the context of the GBF, a foundational principle is national ownership, which recognizes that monitoring systems and data for reporting against international agreements and treaties are nationally owned and driven, reflecting national priorities and knowledge needs. Multiple principles come together in this step.

#### 1.1.1. Adoption of core principles.

There is a need for the formal adoption of principles analogous to IPCC’s TACCC (Transparency, Accuracy, Completeness, Comparability, Consistency), adapted for the complexities of biodiversity monitoring (e.g., accuracy may refer to correct species identification, precise and unbiased population trend estimates, or reliable habitat mapping).

#### 1.1.2. Ethical guidelines.

The BMSF would develop and adopt ethical guidelines covering seven topics inherent to all monitoring activities. The first topic is respect for life and ecosystems which involves minimizing disturbance during data collection and nondestructive sampling wherever possible. The second is ensuring Indigenous Peoples and Local Communities (IPLCs) have Free, Prior, and Informed Consent (FPIC), full and effective participation, and inclusion of traditional knowledge and rights (including data ownership). This goes beyond FAIR to fully embrace CARE principles for Indigenous Data Governance, and equitable benefit-sharing from data and its use (aligned with Targets 21 and 22 of GBF). The BMSF would explicitly state that the purpose of monitoring may be defined by non-Western objectives, such as upholding the dignity of nature. The third is data sovereignty and security which is especially important for sensitive data (e.g., locations of endangered species or sacred natural sites), which some countries, rightsholders, and other organizations may not wish to share data publicly. Fourth is the use of federated learning protocols that have the potential to help actors participate in monitoring while maintaining privacy (30, 31). Fifth, there is a crucial need to specify the transparency of data use prior to data collection, including the intended primary uses of the monitoring data, how it will be shared to contribute to stated monitoring objectives, and any anticipated secondary uses or sharing. This step can address Target 23 of the GBF which requires that monitoring design and implementation consider gender roles and knowledge. Sixth is the use of the precautionary principle to guide monitoring design and interpretation where significant scientific uncertainty exists about threats or effectiveness of interventions. Finally, the seventh topic concerns the sustainability and resilience of monitoring programs to policy shocks and funding interruptions, which are critical given the value of long-term datasets for understanding trends and policy outcomes.

Once the ethical framework for the monitoring program or network has been established, these standards are applied at each step along the monitoring workflow we described below. We describe these modules in turn and suggest what each module requires for implementation.

### 1.2. Step 1: “Sensing and Knowing” Step: Observations and data collection.

This module focuses on the systematic and ethical collection of primary data from multiple evidence streams—including scientific methods and Indigenous Knowledge systems—to assess biodiversity status, trends, and the causes of change.

#### 1.2.1. Activities and standards.

##### 1.2.1.1. Development with indigenous knowledge systems.

Actively create ethical space and culturally appropriate pathways for the respectful inclusion of Indigenous Knowledge (IK), innovations, and practices related to biodiversity monitoring and assessment [e.g., the principle of “Two-Eyed Seeing” (Mi’kmaq: Etuaptmumk, (25)], or the place-based *mātauranga* (the Māori knowledge system), which includes historical knowledge, culturally recognized indicator species, and intuitive, holistic assessments of environmental health. Māori communities recognize different indicators and monitoring approaches, and these differ to scientific monitoring systems (26, 27, 32). Within these worldviews, the environment is a web of relationships to be nurtured.

This includes recognizing that the purpose of monitoring can be defined by non-Western objectives, such as upholding the dignity of nature as a relative, not just tracking resources for human use. Indigenous Peoples are knowledge and rights holders, so participation should recognize their FPIC for any engagement and process. Protocols could be developed collaboratively (with CARE principles) that weave together IK biodiversity concepts with nonindigenous approaches.

Support Indigenous-led monitoring initiatives and ensure equitable benefit-sharing from any coproduced knowledge. The standards in Step 1 should be explicitly designed to accommodate and legitimize multiple evidence streams. The typology of data cannot be limited to quantitative metrics. It should include pathways for respectfully incorporating qualitative, narrative, and place-based knowledge. Standards could include qualitative and narrative data forms inherent in many IK systems (e.g., Indigenous-relevant biodiversity indicators) alongside quantitative scientific data.

##### 1.2.1.2. Indicator-driven design.

Monitoring protocols could be designed to directly inform specific GBF headline, component, complementary, or national indicators (e.g., habitat extent for Target 1, species population trends and genetic diversity for Target 4, protected area coverage and effectiveness for Target 3). This is needed for existing indicators and those under development to be included in the future. At this time, 12% of the elements of the GBF lack an indicator (8). As gaps in indicators are addressed, there is a need to ensure that they are responsive to actions taken to mitigate biodiversity loss. They may also, in some cases, need to be modified to address the needs of other subnational actors (Indigenous Peoples, local communities, regional governments, and businesses).

##### 1.2.1.3. Standardized protocols (a tiered approach).

A tiered approach (like IPCC Tiers for greenhouse gas inventories) allows countries to start with simpler methods and progressively adopt more sophisticated ones as capacity grows. A similar approach primarily but not only directed to businesses is adopted by the Nature Positive Initiative with its associated nature metrics. Development of internationally recognized, yet nationally adaptable, field protocols for “basic” observations used to estimate Essential Biodiversity Variables [EBVs; (21) and Essential Ecosystem Service Variables (EESV; (22, 23)] that are required by indicators used to track progress to some of the Targets of Goal A and Goal B of the KM GBF. Essential Ocean and Essential Climate Variables provide critical complementary information about the system and recognized drivers. Other protocols are required for assessing drivers and impact variables (e.g., socioeconomic data on pressures and benefits: EEIV, Essential Environmental Impact Variables (24).

##### 1.2.1.4. Technology integration.

The first consideration is the sampling strategy with the need for guidance on statistically robust sampling designs (e.g., balanced, stratified random, systematic) appropriate for particular EBV classes (e.g., occurrences, genetic samples, etc.). Standardized methods draw upon remote sensing, including satellite and drone imagery, to estimate indicators of habitat and ecosystem extent, condition, connectivity, and land-use change. In situ technologies require guidance on best practices, such as the use of camera traps, acoustic sensors, GPS tracking, etc., with the methods for standardized calibration and validation, deployment, and data extraction from sensors.

Last there is a need to combine IPLC monitoring and citizen derived data. Protocols are available for working with data from validated citizen science programs and community-based observations and traditional knowledge, ensuring FPIC, ethical engagement and the implementation of FAIR and CARE principles.

### 1.3. Step 2: “Processing and Curation” Step: Data Processing and Management.

This step builds a module that deals with the transformation of raw acquired data into validated, organized, and accessible datasets ready for analysis, ensuring data quality and longevity.

#### 1.3.1. Activities and standards.

##### 1.3.1.1. Data validation and quality assurance/quality control.

This step fosters the use of standardized procedures for data cleaning, error checking, deduplication, outlier identification, and quality assurance/quality control for different data types.

##### 1.3.1.2. Data formatting and interoperability.

There is a need to adopt common data standards and formats (e.g., Darwin Core for species occurrences, standardized metadata schemas like ISO 19115 for geographic information) to ensure interoperability between national systems and global repositories (e.g., GBIF) for particular needs [e.g., Environmental Impact Assessments, (33)]. This interoperability could be operationalized through well-documented, public APIs that allow both humans and machines to submit, query, and retrieve data in a standardized manner, facilitating automated data flows from collection devices and platforms into curation workflows. This combines with protocols for data version control and long-term secure archiving to further ensure interoperability.

##### 1.3.1.3. Multimodal integration.

This involves combining diverse sources (species observations, movements, environmental DNA, habitat maps, bioclimatic variables) into calibrated spatial and temporal datasets of varying granularity [e.g., BioCube, (34)].

##### 1.3.1.4. Database management.

Database management provides guidance on establishing and maintaining biodiversity databases (potentially using open-source platforms) capable of storing diverse data types (spatial, tabular, genetic, media). This activity applies if the focus is national datasets or more local community-based monitoring and information systems.

##### 1.3.1.5. FAIR data principles.

Commitment to making data Findable, Accessible, Interoperable, and Reusable, within the bounds of ethical, commercial, and security considerations (19).

##### 1.3.1.6. Indigenous knowledge.

The standards for data models and ontologies can be flexible. While technical interoperability (e.g., Darwin Core) is important, the framework should also allow for data to be structured and aggregated according to culturally defined relational frameworks, not just Western scientific taxonomies. The data storage can be designed to capture both automated sensor data and insights directly provided by Indigenous communities.

### 1.4. Step 3: The “Trust” Step: Provenance and Licensing.

This module ensures transparency and credibility by meticulously tracking the origin and processing history of data and clearly defining rights and permissions for its use.

#### 1.4.1. Activities and standards.

##### 1.4.1.1. Metadata standards.

Comprehensive metadata capture for all datasets, detailing data collection methods, processing steps, quality assurance and quality control, personnel involved, temporal and spatial coverage, and any constraints to access.

##### 1.4.1.2. Data lineage tracking.

Data systems that track the “chain of safekeeping” for data from collection to final product, allow for data and analytical reproducibility and auditability.

##### 1.4.1.3. Clear licensing.

Adoption of clear data licensing frameworks (e.g., Creative Commons licenses) that balance open access with the need to protect sensitive information or respect IPLC data sovereignty.

##### 1.4.1.4. Attribution.

Standards for proper attribution of data sources are applied when data are reused or integrated (e.g., for life-cycle analysis).

##### 1.4.1.5. Security protocols.

This requires the use of measures to protect sensitive data from unauthorized access or misuse.

##### 1.4.1.6. Indigenous data sovereignty.

Standards can provide explicit guidance on codesigning data access and governance protocols with IPLC partners. It could support tiered access levels and recognize that not all data in a monitoring system will be “open.” The principle of guardianship would be a core concept within this “Trust” step.

### 1.5. Step 4: The “Analysis” Step: Analytical Methods.

This step involves the application of open, discoverable, and reusable scientific and statistical methods to process data to derive meaningful information about biodiversity patterns, trends, and the effectiveness of interventions.

#### 1.5.1. Activities and standards.

##### 1.5.1.1. Standardized analytical approaches.

Standardization does not require use of a single analysis or method but rather agreed-upon—and regularly peer reviewed—workflows reflecting best practices. This standardization is available for common analyses like population trend estimation, species distribution modeling, habitat change analysis, connectivity analysis, and ecosystem condition assessment.

##### 1.5.1.2. Uncertainty quantification.

The quantification of uncertainty is an important part of the assessment and reporting associated with estimates and trends for almost all variables. Reporting uncertainty can be drawn from standards prescribed by IPCC and IPBES (35).

##### 1.5.1.3. Models and software.

This is the implementation of standardized and reproducible approaches for modeling and artificial intelligence, including the documentation of algorithms, and assumptions used [e.g., ODMAP and ROBBIT (36, 37)], and the provenance of AI algorithms (e.g., training set used, training recipe, architecture, DOI for the evaluation of the algorithm). This also involves the promotion of free and open-source analytical software [Open Source Initiative (2007); e.g., software libraries, QGIS, platform for biodiversity analytics such as BON in a Box] and provision of training, although methods could be documented such that they can be implemented independently of specific software choices. Protocols for validating and calibrating models used for extrapolation or prediction or trends are vital for strong inference. This includes creating spatial/temporal training, validation, sensitivity analysis, and training-test data splits for robust model evaluation).

##### 1.5.1.4. Indigenous knowledge.

Standards for “accredited analytical workflows” could be expanded to include methodologies for ILK-informed and trained AI algorithms and machine learning models (38). This implementation of “Two-eyed Seeing” (25) means that AI models see the world through both scientific and Indigenous lenses, potentially revealing insights neither could find alone. The framework should also address the ethical need for AI interpretability and explainability, avoiding “black box” models that are not transparent to the communities using them.

##### 1.5.1.5. Integration of diverse datasets for attribution.

This involves methods for integrating ecological data with socioeconomic data, pressures, and response data to understand drivers and impacts.

##### 1.5.1.6. Gap analysis.

Methods for synthesizing uncertainty, validation, and calibration data to identify priorities for collecting more or different data to improve performance.

### 1.6. Step 5: The “Insight” Step: Indicator Calculation and Interpretation.

This module focuses on translating analytical outputs into indicators (including those required by the GBF), interpreting their meaning in the context of targets, baselines, and ecological understanding.

#### 1.6.1. Activities and standards.

##### 1.6.1.1. Indicator calculation and protocols.

This step promotes open, documented, and repeatable methods for calculating indicators from the analyzed data. These methods could guide aggregation protocols to facilitate assessments and regional and global levels. Crucially, these protocols could be updated to reflect changes to methods and input data, but this review is conducted in Steps 2 to 4.

This step can support the development and use of holistic, composite indicators that can integrate both quantitative and qualitative data. The BMSF could promote Indigenous indicator frameworks which provide a culturally grounded way to interpret the overall “picture” of ecosystem health (38). This moves beyond single-variable trend lines to holistic assessments of well-being.

##### 1.6.1.2. Baseline establishment.

Expert guidance on establishing robust baselines and reference conditions against which progress can be measured is critical.

##### 1.6.1.3. Trend interpretation.

This promotes the adoption of frameworks for interpreting observed trends (e.g., stable, declining, improving) in the context of uncertainty (e.g., due to short, noisy, time series) relative to targets and ecological thresholds (39, 40).

##### 1.6.1.4. Attribution analysis.

This requires the adoption of methods to assess the extent to which observed changes can be attributed to particular direct and indirect drivers, including conservation actions or policies (17, 41, 42). Here, clearly defined counterfactuals are needed for robust attribution (43).

##### 1.6.1.5. Synthesis and narrative development.

Guidance on synthesizing multiple indicators to provide a holistic picture of progress, such as toward the KM GBF Goals and Targets.

### 1.7. Step 6: The “Reporting” Step: Reporting and Disclosure.

This module covers the communication of monitoring results at subnational (i.e., by companies reporting under the CSRD) and national levels. At the national level, a reporting authority submits findings to the CBD Secretariat (as per Article 26) in a consistent and timely manner. This communication is framed against the ambition set out in National Biodiversity Strategy and Action Plans (NBSAPs).

#### 1.7.1. Activities and standards.

##### 1.7.1.1. Standardized reporting formats.

This requires support for the development of common reporting formats and templates, for example, for National Reports to the CBD, aligned with the GBF’s monitoring framework, or for companies reporting under methods of the TNFD or similar disclosure frameworks (in line with Target 15).

##### 1.7.1.2. Public accessibility.

Making reports and (nonsensitive) supporting data publicly accessible through national clearing house mechanisms (maintained by UN CBD) and potentially through biodiversity monitoring portal and dashboard (e.g., UN Biodiversity Lab) following dashboard design standards for data and trend information (44).

##### 1.7.1.3. Verification/review mechanism.

Establishment of a supportive ongoing technical review process (analogous to UNFCCC’s international consultation and analysis) to enhance transparency, credibility, share lessons learned, and identify capacity-building needs.

##### 1.7.1.4. Communication products.

This relates to providing guidance on how to develop diverse communication products to reach different audiences. The standard in this step should provide guidance on developing a wide range of communication products (dashboards, policy briefs, summaries for policymakers, public-friendly reports), including visual, narrative, and interactive platforms that can communicate a relational worldview. Instead of just showing charts, the User Interface could embed stories, audio clips, and images from the Indigenous Knowledge Workspace directly alongside the scientific data, providing a richer, more contextual understanding of what the numbers mean. The goal of reporting is not just to inform, but to connect and reflect the values established Step 0.

### 1.8. Specific Instances of the General BMSF Workflow.

As an illustration of how these steps create an auditable “chain of evidence,” we provide a detailed example of a workflow for an indicator assessing the *Area and connectivity of natural forest ecosystems* is detailed in Table 1. This workflow is relevant to GBF Target 3. This indicator involves diverse methodologies and data streams—from remote sensing to ground-truthing and connectivity modeling—that benefit greatly from the standardization the BMSF provides. The complete, step-by-step application of BMSF standards, including objectives, necessary steps, and evidence for certification at each stage. The application of this chain of evidence ensures that decisions informed by the indicator are based on high-quality, comparable, and scientifically credible information.

**Table 1.** A national agency in “Country X” tasked with reporting one of the (component or complementary) indicators for the change over time in the connectivity of natural forest ecosystems for Target 3 of the GBF

Step in cycle	Objective	Implemented steps	Evidence documents for certification
Step 1: Planning and design of observations and data acquisition	To define precisely the resources invested, what will be measured, and how, ensuring the result is fit-for-purpose recognizing the rights (FPIC) of forest-dependent communities.	Define “Natural Forest” and reference state: The agency formally adopts a national definition based on an international standard [e.g., FAO, (45)], but with specific criteria to exclude plantations (e.g., minimum patch size, species diversity criteria). Define Connectivity Metric: An indicator is chosen [e.g., ProtConn or Integral Index of Connectivity, IIC; (52)] from a peer-reviewed and approved publicly available source that supports and updates the methodology. Reviewed code or software used to calculate it and the counterfactual is specified (e.g., Conefor, BON in a Box). Stakeholder/Rightsholder Engagement: Documented consultations are held with the national forestry department and Indigenous community representatives to validate the “natural forest” map layers and understand data sensitivities.	BMSF S1-Certified Design Evidence: A publicly available “Design and Analysis Plan” with a DOI. Criteria: The document should contain the explicit definitions, the chosen metric, and evidence of stakeholder consultation (e.g., approved by stakeholders and rightsholders). Outcome: Anyone using the final data knows exactly what was intended and what “forest” means in this context, relative to the reference.
Step 2: Data acquisition, processing, and management	To gather the raw spatiotemporal and ground-truth observations and data required for analyses.	Acquire Satellite Imagery: The agency acquires freely available Sentinel-2 Level-2A surface reflectance imagery for the entire country for the years 2020–2025. Acquire Ground-Truth Data: Collate data from the National Forest Inventory (NFI) plots and recent, quality-graded citizen science observations (e.g., research-grade GBIF records of forest-indicator species).	BMSF S2-Certified Acquisition Evidence: A metadata document listing all raw input data sources indicating which abide by data standards. Criteria: Each source should be cited with a persistent identifier (e.g., DOI for NFI data, specific query URL for satellite data). The protocol for collecting the NFI data should be included. Value: Ensures the sources and processes for data acquisition are known and traceable.
Step 3: Data provenance and licensing	To process raw data into an analysis-ready, FAIR and CARE land cover map.	Image Processing: Use a documented, version-controlled script to create cloud-free national mosaics from satellite imagery or available satellite knowledge product. Ensure updates to data layers are described and conducted across time periods. Classification: Train a machine learning model (e.g., Random Forest) using the ground-truth data to classify the mosaic into land cover types, including “natural forest.” The model, its training data, and its accuracy assessment are all saved. FAIR and CARE Publication: The resulting 10m resolution “Natural Forest Map 2025” is published in the national data repository. It has a DOI, rich EML metadata, and ideally a CC BY license. The metadata explicitly describes the CARE principles applied (e.g., data for sacred groves were aggregated to a coarser resolution in the public version as requested by Indigenous partners).	BMSF S3-Certified Data Product Evidence: The publicly accessible, derived land cover map dataset. Criteria: The dataset should have a DOI, a complete metadata file describing its full provenance (including the classification model version), a clear license, and a statement on CARE implementation. Value: Creates a trustworthy, reusable asset (the map) that others can build upon.
Step 4: Analysis and Modeling	To execute the analysis to calculate the area and connectivity values.	Area Calculation: A script calculates the total area of pixels classified as “natural forest.” Connectivity Calculation: The forest map is used as input into the specified software to calculate the IIC index. Uncertainty Quantification: The known classification accuracy from Step 3 (e.g., 92% accuracy) is used to calculate a confidence interval around the final area estimate (e.g., 45,210 km^2^ ± 1,240 km^2^). Reproducibility Package: The entire analysis workflow (code, software environment) is packaged into a Docker container ready for use in BON in a Box or similar platforms.	BMSF S4-Certified Analysis Evidence: A link to a Git repository (e.g., on GitHub) containing the analysis code and the Dockerfile. Criteria: The code should be well-documented. The repository should include instructions to reproduce the exact numerical results, including the uncertainty calculations. Value: Guarantees scientific reproducibility and transparency of the calculations.
Step 5: Indicator calculation and interpretation	Translate the numerical results into a formal indicator product.	Indicator calculation: The final results are compiled into a formal “Indicator Factsheet.” Visualization: The factsheet includes a map of forest cover change and a time-series graph showing the trend in area and connectivity (with uncertainty bands) relative to baseline and reference state. Provenance Statement: The factsheet includes a dedicated section linking back to the DOIs of the certified products from steps 1, 2, 3, and 4, creating a complete, clickable “provenance chain.”	BMSF S5-Certified Indicator Evidence: The final, version-controlled indicator factsheet, with a DOI kept in a national repository. Criteria: The factsheet should clearly state the indicator values with uncertainty relative to baseline and reference state. Include visualizations, and provide a complete, linked provenance chain. Value: Creates a single, trustworthy “answer” that is fully auditable.
Step 6: Synthesis with standards for communication reporting and disclosure	To use the certified indicator for official reporting and to inform national action.	CBD Reporting: The agency submits the value and a link to the Step 5 Certified Indicator factsheet in its 7th National Report to the CBD. Policy Briefing: A summary is used to brief the Ministry of Environment on where connectivity is lowest, suggesting priorities for new ecological corridors. Disclosure: The indicator is featured on a public national biodiversity dashboard.	BMSF S6-Certified Application Evidence: A link to the official national report or policy document where the indicator is cited. Criteria: The indicator should be verifiably used to inform policy or meet an international reporting commitment. Value: Demonstrates that the monitoring effort was used in national reporting and made available to global assessments.

The table illustrates how the BMSF might be implemented to ensure the report on this indicator is of high quality and internationally comparable. Certification of a step may require certification of previous steps that produce necessary information. Some workflows in this table already exist and are used to estimate connectivity.

These workflows represent a resource for other monitoring efforts by participants in the same area or elsewhere in the monitoring network (see *SI Appendix*, Table S1 presenting this network benefit). These assembled workflows can be shared via open platforms and updated [e.g., GEO BON’s BON in a Box (47)], thereby serving the broader community contributing to the implementation of monitoring standards.

## 2. Toward Implementation

We now outline the approach required to implement the BMSF, including key elements and the need for an implementing organization. We draw parallels with the process adopted by the IPCC, WMO, and FAO and other organizations for the development and implementation of international monitoring standards in other domains (Box 1).

### 2.1. A Federated Network Model.

Operationalizing the BMSF can be envisioned through a federated, network model [i.e., a Biodiversity Observation Network (6, 48)], fostering both local site engagement and national (or global) synthesis (Table 1 and *SI Appendix*, Table S1). In this model, the network is comprised of numerous site-level participants (actors)—such as field teams (e.g., academia, consulting companies, government agencies), community monitors, and Indigenous Knowledge holders—responsible for data acquisition and initial curation at their respective locations, guided by BMSF-standardized protocols (Step 1, Step 2) disseminated by a coordinating group following FAIR, CARE, and FPIC principles. These locally generated, standardized datasets and knowledge records then flow into a national or regional hub (e.g., a National Biodiversity Monitoring Agency, a national GBIF node). This national hub undertakes data integration, applies standardized analytical workflows for essential variable derivation (EBV, EESV, EEIV) and indicator calculation (Step 4, Step 5), ensures comprehensive provenance and trust (Step 3), and ultimately produces aggregated assessments and reports for national commitments (Step 6). Crucially, this structure allows for a form of “federated learning”: insights derived from the synthesized national picture can be fed back to refine methods and guide local actions, enhancing the entire network’s effectiveness over time (30). This model does not require all raw local data to be shared. This federated approach underpinned by the BMSF ensures scalability, comparability, and local trust and relevance while building a robust, evolving understanding of biodiversity status and trends.

### 2.2. A Phased and Tiered Approach to Adoption.

An implementation would start with a needs assessment, followed by capacity building focused on foundational modules (e.g., Principles, basic Data Acquisition protocols, Curation standards) and key, easily measurable indicators. Subsequent phases would progressively introduce more complex monitoring techniques and indicators under a tiered approach. The first tier would recognize the value of standards not requiring sophisticated and costly technical and technological capacity. Support to phase in subsequent tiers would focus on building and maintaining human capacity and the adoption of methods and technologies that allow scaling of monitoring effort across a broad set of sectors responding to targets under subnational and national biodiversity strategies and actions plans.

### 2.3. Capacity Building.

This is the cornerstone for supporting adoption. The subregional Technical and Scientific Cooperation Support Centres recently selected by the UN CBD could provide training, technical backstopping, and facilitate South–South cooperation. This will involve training on remote sensing, field survey design, data management tools, analytical software, and reporting as per the cycle (Fig. 1).

### 2.4. Governance.

For monitoring frameworks like REDD+ MRV, a centralized, top–down body like FAO makes sense given the direct link to the UNFCCC and IPCC and the coupling of climate observing systems to carbon stock and emissions models and assessments. However, the multifaceted nature of biodiversity and the existing landscape of key organizations make a federated governance model, with national ownership at its heart, more appropriate and likely to succeed for the BMSF. This model is similar to structures (e.g., the Interagency and Expert Group on SDG Indicators) developed by the UN Statistics Commission for monitoring progress to the SDGs (49). The intent is to build on the progress made to date, which is being achieved via distributed networks and bottom–up modes of governance.

This federated organization [e.g., a Global Biodiversity Monitoring Partnership (GBMP)] would have a mission to collaboratively and inclusively develop, maintain, and promote the global adoption and implementation of the biodiversity monitoring standards framework (BMSF) to enable effective tracking of progress toward the KM GBF via its monitoring framework, as well as toward biodiversity goals encoded in other Multilateral Environmental Agreements. It would also provide the foundation for other users and stakeholders to engage, e.g., businesses, IPLCs, and civil society.

This standards body could oversee six core functions. The first of these functions is the development and maintenance of standards. This would involve the development, review, and periodic updating of the BMSF steps and associated technical guidance through technical working groups. Ensure standards are scientifically robust, practically implementable, and adaptable to national contexts, and promote harmonization and interoperability of methods and data. The second function is capacity building and technical support, with the objective of catalyzing and coordinating capacity-building initiatives (training workshops, webinars, e-learning, technical assistance missions) in collaboration with implementing partners. Develop and disseminate training materials and best practice guides and support the development and dissemination of open-source tools and platforms for biodiversity monitoring (e.g., expanding existing tools or fostering new ones). Additional guidelines will be needed to evaluate capacity to deliver training. The third function is knowledge sharing and supporting communities of practice. The key role of this function is to work with organizations that facilitate a global community of practice for biodiversity monitoring by organizing activities such as joint international conferences, workshops, and webinars. Open access to documents, tools, case studies, and contact points for using the standards would foster uptake. The fourth core function of resource mobilization and coordination advocates for increased investment in national biodiversity observation and monitoring networks based on a needs assessment given the monitoring objectives. Help coordinate funding efforts to support BMSF implementation, avoiding duplication and maximizing impact. Provide guidance to funding agencies, investment banks and other financial investors, and philanthropy on priority areas for investment. The fifth function is alignment and harmonization between the BMSF and other relevant global and regional initiatives (e.g., Sustainable Development Goals monitoring, other MEAs, industry initiatives), which would promote harmonization with existing data standards (e.g., an ISO for biodiversity data) and infrastructures. Lastly, support for the review and assurance of quality is needed to develop guidelines and potentially a roster of experts for a voluntary technical review process for monitoring workflows. This support would extend to national biodiversity monitoring reports, to improve quality, and share lessons (akin to UNFCCC’s International Consultation Analysis or technical assessments).

A federated model offers numerous advantages. It would leverage existing expertise and infrastructure from organizations like GBIF, GEO BON, and IUCN, increasing legitimacy and buy-in by involving a diverse range of rightsholders and stakeholders from the outset. This decentralized structure fosters flexibility, responsiveness, and innovation within a common global framework, allowing the GBMP to act as a crucial orchestrator providing the common language and tools to strengthen global assessments of progress.

## 3. Discussion

The pressing need to halt and reverse biodiversity loss, as articulated by the Kunming-Montreal Global Biodiversity Framework, demands a commensurate improvement in how we monitor, report, and act upon changes in the state of nature (8, 50, 51). The BMSF is firmly grounded in decades of progress in biodiversity science, data management, and conservation practice. Many of its components are already considered best practice within their respective domains, including use of Essential Variables (EBVs, ECVs, etc.) as a quantitative framework for focusing data collection, adherence to FPIC, FAIR, and CARE data principles, the use of Darwin core for interoperability and application of established scientific methods, such as standardized field sampling protocols, remote sensing classifications, and statistical modeling for trend analysis. These elements are not new; the core contribution of the BMSF is as a comprehensive, multistep system to guide the standardization of the entire monitoring workflow. The realization of high-quality monitoring workflows under a BMSF would transform disparate data into actionable, globally comparable knowledge, thereby enabling aggregation of evidence and more effective tracking of progress toward conservation targets set at organizational, national, and global levels.

### 3.1. Strengths and Potential Impacts of the BMSF.

The adoption of the BMSF promises several benefits by directly addressing longstanding gaps that prevent disparate monitoring data from becoming actionable knowledge. Its strength lies in creating a standardized and auditable “chain of evidence” that links field observations to high-level decisions. It formalizes the links between each step—from a raw observation to a national indicator—through explicit provenance tracking, metadata, and versioning (the “Trust” step). There are several ways this creates a level of transparency and reproducibility rarely achieved in biodiversity monitoring at scale.

The BMSF fills the methodological gap between data collection and data aggregation. Currently, data from different projects are often incompatible due to varying field protocols, data formats, and analytical choices. By promoting standardized protocols for data acquisition (Step 1), curation (Step 2), and provenance (Step 3), the BMSF creates interoperable datasets. This enhancement is crucial for national reporting, allowing for the robust aggregation of data from diverse actors—including subnational governments, businesses, and local communities—to generate a credible national picture of biodiversity trends. This is also crucial for understanding broad-scale trends, identifying priority areas for action, and evaluating the collective progress toward the GBF targets. It is vital that Parties have the means to consistently and robustly monitor, report, and verify progress while fostering national ownership, capacity, and global comparability.

The BMSF addresses the analytical gap between observing a trend and attributing its causes (17). Understanding why biodiversity is changing is essential for effective management. The BMSF builds a system of community-vetted, standardized, and sustainable analytical pipelines. This promotes methodological comparability, not just conceptual similarity. By promoting accredited analytical workflows (Step 4) and guidance on attribution analysis (Step 5), the framework supports more rigorous assessments of the drivers of change. This directly informs critical conservation decisions, such as prioritizing investments in policies that mitigate key threats or assessing the effectiveness of specific restoration actions against a clear counterfactual.

The framework’s emphasis on transparency, standardized and traceable protocols, provenance tracking, and uncertainty quantification will build greater trust and confidence in biodiversity assessments by businesses (e.g., via TNFD alignment), investors, and the public. Subnational governments and communities (e.g., municipalities) also require credible monitoring workflows to mobilize funding directed toward protection and restoration of nature and the climate risk mitigation and ecosystem service benefits they receive from (i.e., nature-based solutions).

Last, the proposed tiered structure, coupled with the promotion of open-source tools and capacity-building initiatives, aims to empower a wider range of actors, including those in resource-limited settings, to participate in and benefit from robust monitoring. This inclusivity is vital for ensuring national ownership and for integrating diverse knowledge systems, including those of Indigenous Peoples and Local Communities, as emphasized by the CARE principles and specific ethical guidelines within the BMSF. Furthermore, by streamlining reporting processes and clarifying methodological expectations, the BMSF can lead to greater efficiency and reduce duplication of effort, allowing resources to be focused more effectively. Ultimately, the high-confidence biodiversity insights generated through the BMSF will be critical for supporting adaptive management strategies and achieving tangible global conservation goals.

The BMSF is designed to complement and integrate with other key global initiatives, including the UN’s System of Environmental-Economic Accounting (SEEA), the Natural Capital Protocol, and serves as a robust basis for disclosures under the Taskforce on Nature-related Financial Disclosures (TNFD) and Science Based Targets for Nature (SBTN). For example, the BMSF aligns well with the Accounting for Nature framework, conceptualized by the Wentworth Group of Concerned Scientists (2016), which offers a rigorous, transparent, and verifiable approach to environmental accounting. Grounded in reference ecosystem condition benchmarking, it enables the standardized measurement of biophysical asset condition across various scales. The BMSF, therefore, does not compete with frameworks like CSRD, CSDDD, or TNFD; it underpins and enables them (*SI Appendix*, Table S3).

Generally, the BMSF’s flexibility and grounded approach would enable it to support monitoring to meet the needs of a range of different end-user groups: citizen networks and the significant data they are gathering and contributing (52), governments for their NBSAPs, Indigenous groups to enable them to integrate their data and knowledge systems into national and global structures on their own terms, and businesses, which are both data providers (e.g., the datasets they generate to fulfill regulatory requirements such as Environmental Impact Assessments, as well as those generated to understand their biodiversity impacts), and users—for example, in understanding how their contributions support national and international priorities, and in generating reports for shareholders, investors, and bodies such as TNFD.

### 3.2. Positioning the BMSF within the Landscape of Existing Biodiversity Monitoring Frameworks.

The BMSF complements the existing biodiversity monitoring framework. The GBF’s Monitoring Framework describes the information needs and indicator set for assessing collective progress toward global targets. However, its focus rests primarily on the what of monitoring—headline indicators and national reporting templates—rather than the how: the data pipelines, accreditation mechanisms, and ethical governance required to generate comparable and auditable evidence across diverse monitoring systems. For example, the Biodiversa+ Harmonisation Guide offers a valuable operational layer, defining how field protocols and metadata standards can be aligned across research networks to improve interoperability. It complements the BMSF by providing primarily procedural guidance for data collection rather than a full governance or accreditation framework. The IUCN Protected-Area Monitoring Framework provides a robust approach to adaptive management at the site level, linking monitoring outcomes to conservation decisions, but it is challenging to scale and connect local monitoring results to national or global reporting streams.

The BMSF integrates and extends these partial advances by introducing a federated, multiactor architecture that links ethical principles, standardized data acquisition, accredited analytical workflows, and transparent reporting into a single, auditable chain of evidence. In this way, it translates fragmented, scale-specific efforts into a cohesive set of standards capable of supporting consistent, traceable biodiversity accounting from local observations to global indicators.

In practical terms, the BMSF can operationalize and connect these existing frameworks within a unified monitoring architecture. At the global level, it establishes a standards pipeline through which data generated under other protocols can be transformed into GBF-compatible indicators for national and international reporting. Its federated governance model would allow national focal points to maintain alignment with the GBF indicator hierarchy while supporting regional, local, and Indigenous monitoring initiatives to retain data sovereignty and methodological autonomy. By embedding accreditation and metadata traceability, the BMSF ensures that outputs from distributed monitoring systems meet consistent scientific and ethical benchmarks. Acting as the connecting structure across scales, it links bottom–up innovation and contextual specificity with the top–down coherence demanded by global biodiversity policy.

### 3.3. A Pathway to Implementation.

The BMSF is made tractable through the phased and modular implementation model. A country or organization need not adopt the entire framework at once. It can begin with Tier 1 standards focused on foundational principles and easily measurable indicators. As capacity and resources grow, it can progressively adopt more sophisticated Tier 2 and 3 methods for more complex analyses and EBVs, ensuring the framework is both accessible and aspirational. Crucially, adoption will be facilitated by a commitment to open-source principles for all core components, including analytical workflows and technology platforms (e.g., BON in a Box, UN Biodiversity Lab). This approach lowers financial barriers, fosters a global community of developers and users, and ensures the tools can be transparently validated and adapted for diverse needs.

There are compelling reasons to be confident that historical hurdles can now be overcome. The political and technological landscape has fundamentally shifted, creating a powerful window of opportunity. First, the GBF provides the clear, high-level international mandate for standardized monitoring that was previously lacking. Second, the private sector is now a major driver of action, with frameworks like the TNFD creating unprecedented demand for credible, comparable biodiversity data, unlocking new streams of investment and innovation. Third, the technological barriers have been dismantled; accessible cloud platforms, AI-driven analytics, and open-source tools make the operationalization of a sophisticated framework like the BMSF technically feasible at a global scale. Finally, a growing global recognition of Indigenous rights and knowledge systems provides a more just and effective foundation for the codevelopment of monitoring solutions. It is within this new, synergistic context that the following challenges should be addressed.

A primary hurdle will be achieving broad consensus and sustained commitment from multiple international organizations, regional bodies, national governments, scientific bodies, and other stakeholders. A federated governance model for a GBMP could facilitate this, but navigating diverse interests and ensuring coordinated action will require a dedicated commitment to collaboration.

Resource mobilization is another critical challenge. The development and widespread adoption of the REDD+ MRV system were underpinned by substantial, multiyear financial investments (Box 1). Similar long-term financial commitments from governments, multilateral funds (e.g., Global Environment Facility), and philanthropic organizations will be indispensable for developing BMSF standards, building global capacity, supporting national implementation, and maintaining the necessary technological infrastructure. A key priority for this investment should be the development and long-term maintenance of a core suite of open-source software and tools that operationalize the BMSF workflows. While proprietary solutions may arise, a foundation of free and open-source tools is essential to ensure equitable access, enable transparent peer review of methods, and foster a collaborative community that can sustain and improve the framework’s components over time. Without dedicated funding for this open-source infrastructure, the BMSF risks remaining an aspirational framework rather than an operational reality.

We recognize that monitoring and the mainstreaming of biodiversity data into decisions requires deep involvement of subnational governments, NGOs, business and industry and many other societal actors. A robust system for incentivizing the adoption and use of the BMSF is needed. There are several ways to do this. First, link the standards to the national reporting required by the monitoring frameworks of active multilateral environmental agreements (e.g., GBF, SDGs, CMS). Second, initiatives such as the TNFD could align with the BMSF by requiring private sector disclosures to be based on the guidance offered by this framework. Third, encourage governments to require adherence to standards in publicly funded projects and environmental regulations (e.g., impact assessments). Last, explore the potential for voluntary certification schemes for organizations demonstrating adherence to high-quality monitoring standards.

Capacity development is a cornerstone of the model we propose for the BMSF, particularly for enabling participation from developing countries. Significant regional variation in resources and capacity exists and could be recognized when allocating effort to enabling the BMSF. This requires more than short-term training workshops; it necessitates sustained investment in institutional strengthening, local technical expertise, and infrastructure, tailored to national and regional contexts. A tiered approach allows for progressive engagement, but moving countries up the tiers requires dedicated long-term support. Here, the CBS subregional Technical and Scientific Cooperation Support Centres could play a key role.

Furthermore, balancing the need for standardization with the inherent diversity of species and ecosystems and national monitoring priorities will be a long-term task. Standards could be adaptable enough to be relevant in different contexts without losing the core elements that ensure comparability. The development and maintenance of accredited analytical workflows and tools will also require continuous innovation, technical support, and community engagement to ensure they remain fit-for-purpose and widely accessible. Finally, the governance of the GBMP itself will require careful design to ensure it is efficient, transparent, accountable, and truly representative of its diverse constituents in all parts of the world.

### 3.4. Future Work and the Road Ahead.

This introduction of a BMSF is a call to action. Immediate next steps could focus on initiating pilot implementations of the framework in diverse national and regional contexts, potentially focusing on a subset of GBF targets and indicators (as exemplified in Table 1). These pilots will be crucial for testing the practicality of the proposed linking of standards across the steps, refining the tiered approach, identifying implementation bottlenecks, and demonstrating tangible benefits.

The formal establishment and operationalization of a GBMP is a critical institutional step for implementing the BMSF. This involves securing support from national governments and from key partner organizations and networks designed to provide support. A plan defining its governance structure and securing initial operational funding will also be needed. The GBMP will then need to actively build and nurture a global community of practice around the BMSF, fostering knowledge sharing, collaborative problem-solving, and communication of the advantages to all actors seeking to mainstream the monitoring framework.

Simultaneously, effort is needed to begin the detailed development of specific standards via technical working groups dedicated to each step of the BMSF, engaging relevant expert communities (e.g., GEO BON, IUCN, TDWG, UNEP-WCMC) and technical working groups maintained by partner organizations. This includes elaborating on standardized protocols for translating data into EBVs and EESVs, and then onto indicators, developing suites of accredited analytical workflows, and providing clear guidance on implementing ethical principles. Further research will also be essential. This includes developing methodologies for assessing the performance and impact of the BMSF itself, ensuring that the investment in standardization leads to demonstrably better conservation outcomes. Investigating innovative financing mechanisms, including how robust monitoring could provide powerful incentives for investment in nature restoration.

The BMSF will require significant investment of time and resources. However, the urgency of global biodiversity declines and the proximity of GBF targets mean we cannot afford a slow, sequential adoption. Fortunately, a powerful window of opportunity exists to catalyze this process through a strategic “grand collaboration” between the scientific community, public bodies, the private sector, and, critically, IPLC groups. This will complement existing frameworks and standards like SBTN which have already built standardized approaches to driving credible science-based action and TNFD’s framework for guiding biodiversity disclosures. A groundswell of private sector investment is now being directed toward biodiversity monitoring, sparked by GBF Target 15 (*Businesses assess, disclose, and reduce biodiversity-related risks and negative impacts*) and driven in part by new regulatory frameworks, green financing, and market-based imperatives such as the emerging biodiversity credit economy. We believe that this momentum could be channeled and leveraged to drive the formation of public–private partnerships to align corporate monitoring efforts with the standards and principles of the BMSF. Such a collaboration would enable the private sector to invest with confidence, knowing monitoring will be credible and comparable, while simultaneously providing the scientific and conservation communities with the resources, technology, and scalable implementation pathways needed to make the BMSF a reality—particularly in countries currently lacking sufficient capacity. By creating a standardized pathway from private-sector action to nationally aggregated data, the BMSF allows corporations to demonstrably align their nature-related reporting with the indicators of the GBF, and by consistent use of standardized methods for aggregation of evidence allow measurable assessment of the contribution of corporations to national and international goals.

## 4. Conclusion

The Biodiversity Monitoring Standards Framework proposed here offers a structured pathway to address the longstanding challenges of fragmentation and inconsistency in biodiversity monitoring. The BMSF could enable credible comparison of findings by promoting consistent data capture, quality assurance, and validated analytical pathways with uncertainty reporting. This framework would empower local actors, streamline national reporting to the Convention on Biological Diversity, enhance corporate accountability, and ultimately provide high-confidence biodiversity insights for adaptive conservation management. The implementation of the BMSF can be guided by a federated partnership, drawing lessons from established successes in the biodiversity standards community. The BMSF will be essential if the global community is to effectively mount a response that is appropriately scaled to track progress toward the KM GBF targets. The journey from disparate data to decisive, evidence-based action requires a shared commitment to building this common language for understanding biodiversity change and how action can be implemented most effectively to “bend the curve” of biodiversity (53). We hope the global biodiversity community will embark on this collaborative endeavor.

## Supplementary Material

Appendix 01 (PDF)

## Data Availability

There are no data underlying this work.
