# Supplementary material for: From data to decisions: Toward a Biodiversity Monitoring Standards Framework
Source: Proc Natl Acad Sci U S A. 2026 Mar 4;123(10):e2519347123. doi: 10.1073/pnas.2519347123 (PMC12974509; doi:10.1073/pnas.2519347123)
Supplement: Supplementary file 1 — Appendix 01 (PDF) [file pnas.2519347123.sapp.pdf]

## Supporting Information for

# From Data to Decisions: Towards a Biodiversity Monitoring Standards Framework

Andrew Gonzalez, Tom August, Sallie Bailey , Kyle Bobiwash, Philipp H. Boersch-Supan, Neil D Burgess, Barnabas H. Daru, Chris S Elphick, Robert P. Freckleton, Winifred F. Frick, Alice C. Hughes, Nick J. B. Isaac, Julia P G Jones, Marco Lambertini, Oisin Mac Aodha, Anil Madhavapeddy, EJ Milner-Gulland, Andy Purvis, Nick Salafsky , William J. Sutherland, Iroro Tanshi, Varsha Vijay, Sarah Hollis Woodard, David R. Williams

**Corresponding author:** Andrew Gonzalez, andrew.gonzalez@mcgill.ca

**This PDF file includes:** Supporting Tables S1, S2, S3 and S4

**Table S1:**

An example of how implementing the BMSF at local and national levels allows aggregation of learning across sites. This requires supporting local needs and monitoring activities and, where permitted, to support information flows to national monitoring knowledge services. The BMSF, through its emphasis on standardization, facilitates a process analogous to "federated learning" by enabling robust aggregation and synthesis of information without necessarily centralizing all raw data processing in its entirety.

*Standardized inputs*: Because all local sites use standardized field protocols (Step 1) and data formats (Step 2), the data they submit to the National Data Hub are comparable and interoperable. This data flow could be mediated by standardized APIs, allowing diverse local systems (from mobile apps to sensor networks) to programmatically push data to the national hub. Metadata standards (S3) ensure each dataset is well-described, allowing local sites to understand its context and quality.

*Consistent EBV Derivation*: The national hub can apply peer-reviewed analytical workflows (Step 4) to either the site-specific curated data or to aggregated datasets. For example, if each site provides data on tree density and species, local sites can calculate site-level "Habitat Structure" or "Community Composition" EBV components. These site-level EBV "summaries" or "models" can then be aggregated. Alternatively, all raw data points (if shared) can be pooled at the national level to train a single national model for an EBV (e.g., a national species distribution model for a key mangrove species).

*A federated model can be applied for indicator calculation* (Step 5): Local Calculations, Global Synthesis: Site-level teams could potentially calculate certain local indicators or EBV summaries based on their data, using BMSF-standardized methods. These pre-processed results (like local model parameters or indicator values, rather than all raw data) could then be sent to the national body for aggregation.

*Central Aggregation*: The national layer aggregates the site-level EBV/EESV and/or indicator summaries to produce a national picture. For example, the national "trend in forest extent" would be the sum of changes across all sites, each assessed using comparable remote sensing and ground-truthing standards.

*Distributed Learning and Refinement*: The national hub analyzes the aggregated picture and identifies patterns, conservation successes or failures (e.g., "restoration technique X appears most effective in region Y," or “indicator threshold Z is too sensitive/insensitive").

This "learned" information is then disseminated back to all local sites. This might take several forms such as updated monitoring protocols (Step 1 data collection). Recommendations for improved local

management based on national trends. Local site teams can then adapt their local practices based on this centrally synthesized, but locally derived, learning.

Privacy/Sovereignty (Implicit in Step 0, Step 3): While this example has data flowing to a national hub, a true federated model could, in some instances, allow insights to be generated without raw data leaving the local "custodian" (site level). Local participants could run standardized analyses and only share anonymized or aggregated results/model parameters. The BMSF's emphasis on provenance and data use agreements (Step 0, Step 3) would govern this.

| | |
|---|---|
| **Overall Monitoring Objective (S0)** | To assess the status and trends of an ecosystem (e.g., forest, mangrove) nationally, identify areas of degradation, and evaluate the effectiveness of restoration interventions, aligned with National Biodiversity Strategy and GBF targets (e.g., Target 1 for ecosystem area, Target 2 for restoration, Target 4 for species within mangroves). |
| **Monitoring layer 1** | **Local / Site-Level Monitoring Teams & Communities** |
| **Actors** | **Field Teams (NGOs, University Research Groups, Local Community Monitors, Park Rangers):** Multiple teams responsible for distinct ecosystem sites or regions along the coastline.<br><br>**Remote Sensing Analysts (Potentially centralized or regional):** Processing satellite imagery for ecosystem extent and condition. |
| **BMSF Application at Site Level** | **Step 0 (Ethics - Localized):** Adherence to national ethical guidelines, obtaining local community consent (FPIC) if applicable for site access or co-implemented using incorporating local knowledge. Clear communication of local data use.<br><br>**Step 1 (Sensing & Knowing):**<br><br>**Field Teams:** Execute standardized field protocols for selected EBVs (e.g., EBV_EcosystemExtent via ground-truthing, EBV_CommunityComposition via quadrat counts of ecosystem species, crab populations, bird surveys; EBV_EcosystemStructure e.g. canopy height, tree density). Use standardized methods for equipment.<br><br>**Indigenous/Local Knowledge Holders:** If involved, share observations on changes in ecosystem condition, material ecosystem services (realized ecological supply EESV), or traditional uses, using co-developed protocols.<br><br>**Remote Sensing Analysts:** Acquire and pre-process satellite imagery (e.g., Sentinel, Landsat) according to agreed national standards (e.g., specific bands, cloud masking).<br><br>**S2 (Curation - Initial):** |

| | |
|---|---|
| | **Field Teams:** Perform initial data entry into standardized digital forms/databases, basic quality assessment (checking for outliers, completeness).<br><br>**Remote Sensing Analysts:** Georeferenced imagery, perform atmospheric correction.<br><br>**S3 (Trust - Initial):**<br><br>**Field Teams:** Generate basic metadata for their collected field datasets (who, what, when, where, how).<br><br>**Remote Sensing Analysts:** Document imagery sources and pre-processing steps. |
| **Outputs from Layer 1:** | Site-specific observations and curated datasets (e.g., plot data, species lists, local ecosystem extent maps from field data, pre-processed satellite scenes for their area of responsibility). |
| **Layer 2:** | **National / Central Coordinating & Synthesis Hub** |
| **Actors** | **National Biodiversity Monitoring Agency (NBMA) / Lead Research Institution:** The central coordinating body.<br>**Data Management & IT Team (within NBMA):** Manages central database, IT infrastructure.<br>**EBV & Indicator Specialists (within NBMA or contracted experts):** Experts in deriving EBVs and calculating national indicators.<br>**Policy Analysts & Communicators (within NBMA):** Translate findings for policymakers and public. |
| **Responsibilities & BMSF Application at National Level** | **Step 0 (Ethics, Monitoring Principles):** Establishes national ethical guidelines, data sharing policies, and MOUs with local actors. Ensures overall program alignment with national and international commitments. Defines overall purpose specification.<br><br>**Step 1 (Sensing & Knowing - Coordination & Standards Adopted):**<br><br>Develops and disseminates the standardized field protocols and remote sensing processing guidelines used by Layer 1.<br><br>Provides training and capacity building to Layer 1 teams.<br><br>May directly manage national-scale remote sensing acquisition.<br><br>**Step 2 (Curation - Centralized):** |

| | |
|---|---|
| | Receives datasets from all Layer 1 teams.<br><br>Performs data de-duplication, integration, harmonization (e.g., resolving taxonomic inconsistencies, aligning spatial data).<br><br>Manages the national biodiversity database.<br><br>**Step 3 (Trust - Centralized & Aggregation):**<br><br>Creates comprehensive metadata for aggregated national datasets.<br><br>Manages data licensing for national products.<br><br>Ensures provenance tracking from local collection to national product.<br><br>**Step 4 (Analysis - Centralized):**<br><br>**EBV Derivation:** Uses curated data from all sites to generate national-scale EBV products (e.g., National Mangrove Extent Map, National Mangrove Species Richness Trends, National Mangrove Canopy Height Change Map). This involves applying accredited analytical models and workflows.<br><br>**Indicator Calculation (National):** Aggregates site-level information or uses national EBV products to calculate national-level indicators for ecosystem status.<br><br>**Step 5 (Indicator Calculation & Interpretation - National):**<br><br>Calculates national indicators for GBF reporting (e.g., total area of ecosystem, trend in status of ecosystem, number of degraded sites under active restoration).<br><br>Interprets these indicators against national targets and baselines.<br><br>Conducts attribution analysis (e.g., linking ecosystem loss to specific drivers like deforestation, fire, disease, or linking recovery to restoration efforts).<br><br>**Step 6 (Reporting & Disclosure - National & International):**<br><br>Prepares national reports for the CBD and other relevant bodies.<br><br>Develops policy briefs for national decision-makers.<br><br>Publishes national state of ecosystem (e.g., forest, mangrove) reports and makes data/indicators publicly accessible (e.g., via a national biodiversity portal).<br><br>**Adaptive updating of monitoring activities:** |

| | |
|---|---|
| | Assesses overall program effectiveness, identifies data gaps, evaluates EWI performance.<br><br>Uses insights to refine monitoring objectives, protocols (Step 1), analytical methods (Step 4), and indicator thresholds for the next cycle. This feedback flows back to Layer 1. |

**Table S2:** Typology of the key terms used in this paper to describe the BMSF

| Class of component | Definition |
|---|---|
| **Actor/Agent** | An individual, organization, or system performing a Process/Activity. |
| **Data entity** | Observation Record (e.g., a single species sighting, a plot measurement)<br>Indigenous Knowledge Record (e.g., a documented element of IK)<br>Variable Value (a measurement or derived value for a variable) - Metadata Element |
| **Essential variable** | Essential Biodiversity Variable (EBV)<br>Essential Ecosystem Service Variable (EESV)<br>Essential Ocean Variables (EOVs)<br>Essential Climate Variables (ECVs)<br>Essential Environmental Impact Variables (EEIV)<br>Driver Variable (e.g., climatic data, soil type, human pressure data) |
| **Ethical Consideration** | A principle guiding conduct (e.g., CARE, FPIC). |
| **Information Product** | The tangible output at various stages (e.g., raw dataset, curated dataset, EBV layer, indicator value, report). |
| **Method/Protocol** | A specific, documented way of performing a Process/Activity. |
| **Monitoring Objective** | The knowledge need for conservation or policy goals driving the monitoring. |
| **Process/Activity** | An action taken within a BMSF step or module (e.g., data collection, data validation, model fitting, report generation). |
| **Quality Criterion/Metric** | A standard or measure used to assess an Information Product or Process. |
| **Tool/Software/Instrument** | A physical or digital implement used in a Process/Activity. |

**Table S3: the BMSF does not compete with frameworks like CSRD, CSDDD, or TNFD; it underpins and enables them.**

| Framework | Primary Role | Relationship with BMSF |
|---|---|---|
| **CSRD** | Mandates *what* should be reported on biodiversity. | The BMSF is the **Data Engine**, providing the standardized "how-to" for generating the credible, auditable data required by CSRD reports. |
| **CSDDD** | Mandates the *process* of identifying and mitigating impacts. | The BMSF is the **Operational Toolkit**, providing the monitoring workflows needed to both identify impacts (due diligence) and verify the effectiveness of mitigation actions. |
| **TNFD** | Provides a framework (i.e., LEAP) for how to frame and disclose nature-related issues. | The BMSF is the **Measurement Arm**, providing the methods to gather and analyze the robust data needed for the "Evaluate" and "Assess" steps of the LEAP approach. |
| **SBTN** | Provides a framework for *what to aim for* (setting science-based nature targets). | The BMSF is the **Progress Tracker**, providing the standardized monitoring workflows needed to reliably track progress against SBTN targets over time. |

**Table S4: The table of acronyms used in the main text of the paper.**

| Acronym | Full name |
|---|---|
| **BIP** | Biodiversity Indicators Partnership |
| **BON** | Biodiversity Observation Network |
| **BMSF** | Biodiversity Monitoring Standards Framework |
| **CARE** | Collective Benefit, Authority to Control, Responsibility, and Ethics data management principles |
| **UN CBD** | United Nations Convention on Biological Diversity |
| **CMS** | Convention on Migratory Species |
| **CSRD/CSDDD** | Corporate Sustainability Reporting Directive/ Corporate Sustainability Due Diligence Directive |
| **EBV** | Essential Biodiversity Variables |
| **ECV** | Essential Climate Variables |
| **EESV** | Essential Ecosystem Service Variables |
| **EEIV** | Essential Environmental Impact Variables |
| **EOV** | Essential Ocean Variables |
| **FAIR** | Findable, Accessible, Interoperable, and Reusable data management principles |
| **FAO** | Food and Agriculture Organization of the United Nations |
| **FPIC** | Free, Prior, and Informed Consent |
| **GBIF** | Global Biodiversity Information Facility |
| **GBMP** | Global Biodiversity Monitoring Partnership |
| **GEF** | Global Environment Facility |
| **GEO BON** | Group on Earth Observations Biodiversity Observation Network |
| **ICA** | International Consultation and Analysis process (of the UNFCCC) |
| **IIC** | Integral Index of Connectivity |

| | |
|---|---|
| **IPLC** | Indigenous Peoples and Local Communities |
| **IUCN** | International Union for Conservation of Nature |
| **KM GBF** | Kunming-Montreal Global Biodiversity Framework |
| **MEA** | Multilateral Environmental Agreement |
| **MRV** | Measurement, Reporting, and Verification framework |
| **NBSAP** | National Biodiversity Strategies and Action Plans |
| **NFI** | National Forest Inventory |
| **REDD+** | Reducing Emissions from Deforestation and Forest Degradation |
| **SDG** | Sustainable Development Goals |
| **SEPAL** | System for Earth Observation Data Access, Processing and Analysis for Land Monitoring |
| **TACCC** | Transparency, Accuracy, Completeness, Comparability, Consistency |
| **TNFD** | Taskforce on Nature-related Financial Disclosures |
| **TDWG** | Biodiversity Information Standards (formerly Taxonomic Databases Working Groups) |
| **UNFCCC** | United Nations Framework Convention on Climate Change |
| **UNEP-WCMC** | United Nations Environment Programme World Conservation Monitoring Centre |
